# Overcoming distance: an exploration of current practices of government and charity-funded critical care transport and retrieval organizations

**DOI:** 10.1186/s13049-023-01125-6

**Published:** 2023-10-03

**Authors:** Adam Greene, Pierre-Marc Dion, Brodie Nolan, Rob Trachter, Erik Vu, Jan Trojanowski

**Affiliations:** 1 British Columbia Emergency Health Services, Provincial Health Services Authority, Victoria, BC Canada; 2 School of Medicine, Cardiff University, Cardiff, Wales UK; 3 Faculty of Medicine, University of Ottawa, Ottawa, ON Canada; 4 Clinical Epidemiology Program, The Ottawa Hospital Research Institute, Ottawa, ON Canada; 5 Department of Emergency Medicine, St. Michael’s Hospital, Unity Health Toronto, Toronto, ON Canada; 6 Division of Emergency Medicine, Department of Medicine, University of Toronto, Toronto, ON Canada; 7 Department of Emergency Medicine, Nanaimo Regional General Hospital, Nanaimo, BC Canada; 8 Division of Emergency Medicine, Department of Medicine, Vancouver General Hospital, University of British Columbia, Vancouver, BC Canada; 9 Division of Critical Care Medicine, Department of Medicine, Vancouver General Hospital, University of British Columbia, Vancouver, BC Canada; 10 Division of Critical Care Medicine, Department of Medicine, Kelowna General Hospital, University of British Columbia, Kelowna, BC Canada

**Keywords:** Critical care, Transport, Retrieval, Aeromedical

## Abstract

**Background:**

For critically ill and injured patients, timely access to definitive care is associated with a reduction in avoidable mortality. Access to definitive care is significantly affected by geographic remoteness. To overcome this disparity, a robust critical care transport (CCT) or retrieval system is essential to support the equity of care and overcome the tyranny of distance. While critical care transport or retrieval systems have evolved over the years, there is no universally accepted system or standard, which has led to considerable variation in practices. The objective of this mixed-methods study was to identify and explore the current clinical, operational, and educational practices of government and charity-funded critical care transport and retrieval organizations operating across access- and weather- challenged geography.

**Methods:**

This study utilized a mixed-methods approach comprising a rapid review of the literature and semi-structured interviews with identified subject matter experts (SME).

**Results:**

A total of 44 articles and 14 interviews with SMEs from six different countries, 12 different services/systems, and seven operational roles, including clinicians (physician, paramedic, and nurse), educator, quality improvement, clinical governance, clinical informatics and research, operations manager, and medical director were included in the narrative analysis. The study identified several themes including deployment, crew composition, selection and education, clinical governance, quality assurance and quality improvement and research.

**Conclusion:**

This mixed-methods study underscores the paucity of literature describing current clinical, operational, and educational practices of government or charity-funded CCT or retrieval programs operating across access- and weather- challenged geography. While many common themes were identified including clearly defined mission profiles, use of dedicated or specialized transport teams, central coordination, rigorous selection processes, service-sponsored graduate education, and strong clinical governance, there is little consensus and considerable variation in current practices. Further research is needed to identify and harmonize best practices within the CCT and retrieval environments.

**Supplementary Information:**

The online version contains supplementary material available at 10.1186/s13049-023-01125-6.

## Background

For critically ill and injured patients, timely access to definitive care is associated with a reduction in avoidable mortality [1–8]. Access to definitive care is significantly affected by geographic remoteness [9, 10]. This is particularly evident in large and sparsely populated countries such as Australia and Canada, where health care is highly regionalized, with secondary-, tertiary- and quaternary-level care concentrated into regional hubs. To overcome this disparity, a robust critical care transport or retrieval system is essential to support the equity of care and overcome the tyranny of distance.

However, the transport of critically ill or injured patients is often perilous and fraught with clinical, operational, and logistical challenges [11], as the transport environment is both unpredictable and unforgiving. Some specific challenges include high patient acuity, physiologic stressors of flight, long distance and inclement weather. As a result, critical care transport (CCT) or retrieval programs must work collaboratively with various healthcare partners to resuscitate, stabilize, and provide highly specialized primary and secondary transport of critically ill and injured patients to definitive care [12, 13]. While CCT or retrieval systems have evolved over the years, there is no universally accepted system or standard, which has led to considerable variations in practices [12, 13].

## Objective

The objective of this mixed-methods study was to identify and explore the current clinical, operational, and educational practices of government and charity-funded critical care transport and retrieval organizations operating across access- and weather- challenged geography.

## Methods

### Study design

This study utilized a mixed-methods approach comprising a rapid review of the literature to identify and examine current clinical, operational, and educational practices and to identify subject matter experts (SME) and semi-structured interviews with identified SMEs. This study adhered to the quality assurance and quality improvement standards outlined in Tri-council Policy Statement Article 2.5 and was exempt from a formal research ethics board review as confirmed by the University of British Columbia Office of Research Ethics. Informed consent to participate in this study was obtained from all participants.

### Setting

Government or charity funded CCT and retrieval organizations are defined as organizations whose main funding source is government revenue or charitable donations. Operating across access- and weather- challenged geography is defined as an organization whose response area includes one or more of the following challenges: rural/remote population, mountainous regions, maritime weather, or temperature extremes.

### Literature review

A search of the CENTRAL, MEDLINE, EMBASE, and PubMed databases was conducted in consultation with a medical librarian for all literature published between January 1, 2003, and March 1, 2023. Keywords were defined a priori by reviewing the medical subject headings (MeSH) terms of articles identified in preliminary literature searches and the Commission for the Accreditation of Air Medical Transport Systems (CAMTS) and the European Aero-Medical Institute (EURAMI) standards and combined using the Boolean operator ‘OR’ before to applying ‘AND’ to structure the search. A detailed search strategy is outlined in Additional file 1: Appendix. The search was repeated three times during the review period. The inclusion and exclusion criteria are presented in Table 1. Additional manual searches of reference lists and grey literature (government reports, professional documents and academic papers not controlled by commercial publishers) were also conducted in line with the strategy proposed by Goldin et al. [14]. The literature review aimed to identify major themes related to current clinical, operational, and educational practices and subject matter experts. The quality of the information was not assessed.

**Table 1:** Inclusion and exclusion criteria

Inclusion	Exclusion
English language	Languages other than English
Published from 2005 onwards	Published prior to 2005
Government or charity funded programs	Private, for-profit, or military funded programs
Primary operation across access- and weather- challenged geography	Limited or no operation across access- and weather- challenged geography
Articles that describe clinical, operational, and educational practices	

### Data extraction and analysis

The authors utilized grounded theory to identify and extract key terms, phrases, and concepts from the data sets. This data collection process and open coding analysis allowed for the emergence of themes that informed subsequent data collection and analysis [15]. To further expand and refine the codes, a constant comparative method was used [16–19]. Journaling and margin notes were also employed to inform the coding structure, which was reviewed and integrated into the emerging data set. The authors continued this process until data saturation, but postponed finalizing analyses until the literature and interview data sets could be combined.

### Semi-structured interviews

Following a review of the literature, semi-structured interviews were conducted with SMEs. Most identified systems were in Australasia, Canada, and Northern Europe. Given the diversity of CCT programs in these areas, the sampling strategy was designed to ensure broad representation both in geography and professional breadth while using saturation of ideas as an endpoint.

### Participants

Subject matter experts were identified, recruited, and enrolled using purposive and snowball sampling strategies. A nomination strategy was developed to identify those recognized by the community to be most suited to speak on its behalf and ensure broad representation in both geography and professional breadth. In addition, participants were asked whom should be interviewed. Any nominations were cross-referenced against the existing pool and sampling goals and participants were added as necessary.

### Interview guide

All interviews were semi-structured, and most were two-to-one. The interview guide was developed after identifying themes from the literature and targeted (a) demographics, (b) deployment, (c) crew composition, (d) selection and education, (e) scope of practice, (f) clinical governance, (g) quality assurance and quality improvement, and (h) research. The interview questions were open-ended in nature with probing questions used where necessary. Prior to data collection, the interview questions were piloted internally and revised to ensure clarity and impartiality. The interview guide remained open to revision throughout the data collection phase to allow for discussion of issues raised in earlier interviews.

### Data collection and management

All the interviews were conducted by the authors. Consent was obtained in writing and verbally prior to each interview. Ten interviews were conducted over video conference and recorded, while two interviews were conducted over the telephone and could not be recorded due to technical issues. All interviews were allowed to end naturally. Following each interview, any recordings were reviewed, and the summaries were coded by the interviewing author(s). All summaries were verified for accuracy prior to analysis. The data was stored, organized, and analyzed using Microsoft Excel for Mac (Microsoft Corporation, version 16.73).

### Data analysis and interpretation

All summaries were coded primarily by AG, with a subset additionally coded by RT and JT. Open coding was used to identify areas requiring additional data or new lines of inquiry. The authors used a constant comparative method to refine codes and synthesize data into meaningful groups until themes emerged. The initial analyses and coding of the literature and semi-structured interviews were merged into a single data set for further analysis. This merger allowed each data set to be treated equally and considered in relation to each other. The authors met several times to analyze the data, to identify themes, and to convert these themes into narrative text.

## Results

**Fig. 1:**
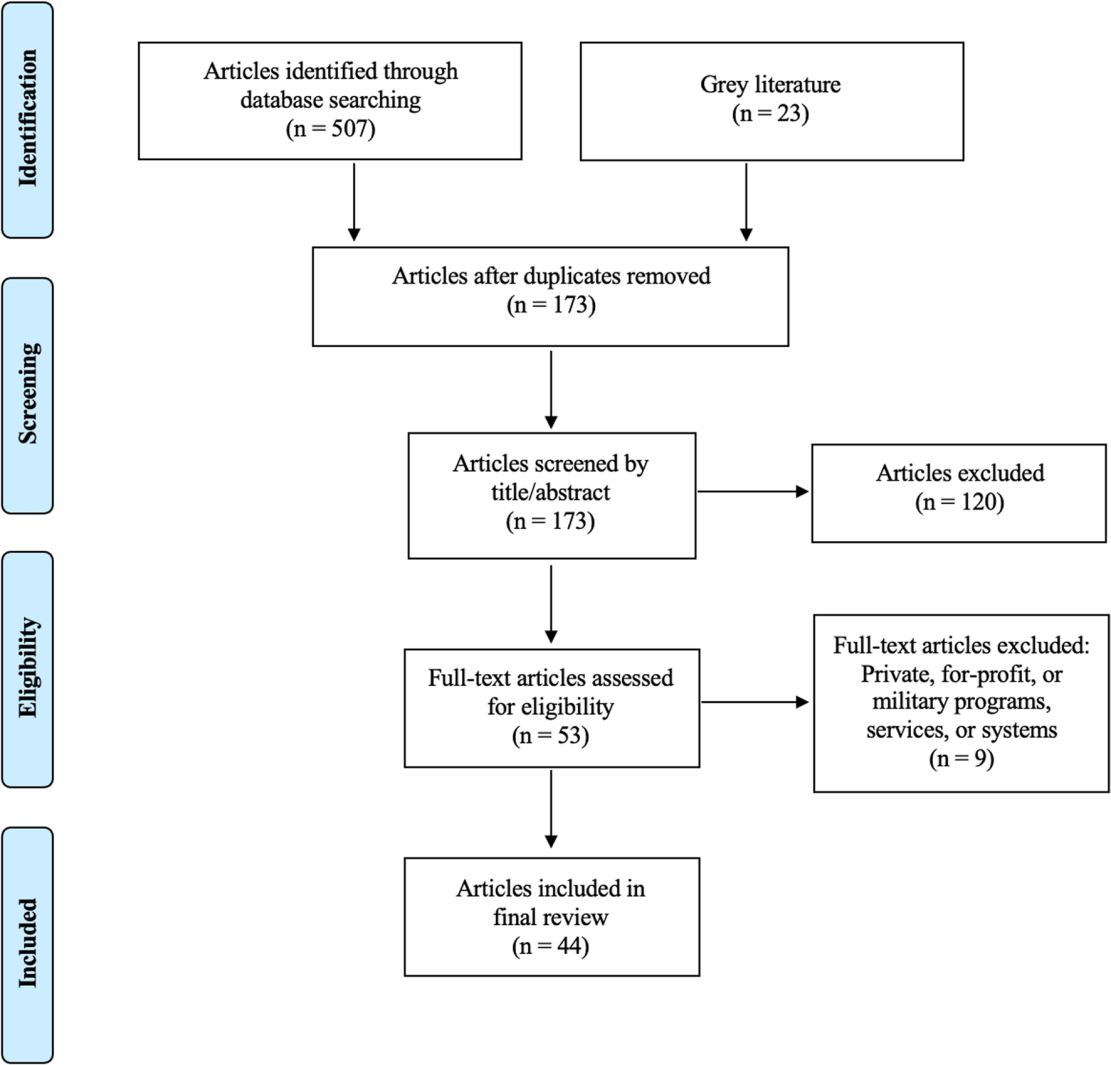
Flowchart of article selection for literature review

The literature search yielded 507 articles (Fig. 1). Following the removal of 334 duplicates, the titles, and abstracts of 173 articles were screened according to the inclusion and exclusion criteria. Twelve additional articles were identified through hand searching and backward chaining. After full-text reviews, 44 articles were included. The characteristics of included studies are shown in Table 2. Most studies were observational and conducted in Australasia or Northern Europe. Fourteen SMEs were interviewed from six different countries, 12 different services/systems, and seven operational roles. The characteristics of SMEs are shown in Table 3. Seven broad themes were identified including deployment, crew composition, selection and education, clinical governance, quality assurance and quality improvement, and research.

**Table 2:** Articles included in the literature review

Type of study	References	Location	Subject of study
Systematic review	Laverty et al. [38]	Canada	Primary aeromedical retrieval crew composition
	Masterson et al. [46]	Various	Clinical crew competencies in helicopter emergency medical services (HEMS)
	von Vopelius-Feldt et al. [34]	England	Critical care paramedics: Where is the evidence? A systematic review
Scoping review	Muyambi et al. [61]	Various	Aeromedical retrieval services characteristics globally: a scoping review
	Mathew et al. [26]	Various	Optimising medical retrieval processes and outcomes in remote areas in high-income countries
	Edwards et al. [54]	Australia	Scoping review of air ambulance outcome measures
Literature review	King et al. [62]	Australia	Primary aeromedical retrievals in Australia
Observational	Bredmose et al. [49]	Various	An international comparison of independently developed training courses
	Parenmark and Walther [30]	Sweden	Risks associated with intensive care unit-to-unit capacity transfers
	Tommila et al. [57]	Finland	Standardised data collection in prehospital critical care
	Denton et al. [36]	England	Evaluation of advanced critical care practitioner-led inter-hospital transfers
	Franklin et al. [25]	Australia	Aeromedical retrievals in Queensland Australia over five years
	Jagtenberg et al. [31]	Norway	Norwegian Air Ambulance base locations
	Lyons et al. [43]	Wales	Impact of a physician-led critical care on trauma survival in Wales
	Garwood et al. [24]	Australia	Aeromedical retrievals in Western Australia over five years
	Grier et al. [29]	England	Analysis of 1124 critical care transfers in an English critical care network
	Maddock et al. [42]	Scotland	Physician-led HEMS in Scotland
	Saviluoto et al. [56]	Finland	The first seven years of HEMS in Finland
	Alstrup et al. [23]	Denmark	Characteristics of HEMS patients in Denmark over five years
	Alstrup et al. [55]	Denmark	The Danish HEMS database
	Hepple et al. [45]	Wales	Physician-led HEMS in Wales
	Moors et al. [40]	Netherlands	Physician-led HEMS in Netherlands
	Oud et al. [58]	Australia	Long-term effectiveness of the airway registry at Greater Sydney Area HEMS (GSA-HEMS)
	Smith et al. [41]	England	Prehospital analyses of northern trauma outcome measures: PHANTOM
	Kornhall et al. [63]	Sweden	HEMS in rural Sweden
	Delorenzo et al. [22]	Australia	Fixed wing transports in Victoria Australia over five years
	Haugland et al. [53]	Norway	Developing quality indicators for physician-led HEMS
	Kennedy et al. [50]	Australia	Clinical governance and workforce models in prehospital and retrieval medicine
	Funder et al. [64]	Denmark	Long-term follow-up of trauma patients before and after implementation of HEMS
	Kennedy et al. [12]	Australia	Impact of integrated adult retrieval service on major trauma outcomes
	Andrew et al. [21]	Australia	Characteristics of HEMS patients in Victoria Australia
	von Vopelius-Feldt and Benger [34]	England	Who does what in prehospital critical care?
	Hesselfeldt et al. [44]	Denmark	Impact of a physician-staffed helicopter on a regional trauma system
	Margolus et al. [20]	Australia	Twelve years of experience with Royal Flying Doctor Service (RFDS)
	Corfield et al. [39]	Scotland	Rural EMRS in Scotland: The first year
	Whitelaw et al. [65]	Scotland	Establishing a rural EMRS in Scotland
Grey	Rawlinson et al. [52]	Wales	EMRTS Cymru service evaluation
	Alberta Health [66]	Canada	HEMS services report
	Ornge [51]	Canada	Quality improvement plan
	Wiggin [48]	Australasia	Learning from the paramedic-led critical care teams in Australasia
	Jashapara [32]	England	An introduction to critical care paramedics in the United Kingdom
	Jashapara [47]	England	Delivering enhanced pre-hospital critical care: a cost-effective approach
	NHS Scotland [67]	Scotland	ScotSTAR strategic vision
	Boyle et al. [68]	Scotland	Evaluation of the EMRS in Scotland

**Table 3:** Characteristics of SMEs

Location	Organization	Background	Role
Australia	Air Ambulance Victoria	Paramedic	Quality improvement
Australia	Greater Sydney Area-HEMS	Physician	Clinician/Medical manager
Australia	Retrieval Services Queensland	Physician	Medical director
Australia	RFDS Queensland	Nurse	Clinical governance
Canada	BCEHS	Paramedic	Clinician/Educator
Canada	Ornge	Physician	Clinical governance
Canada	EHS LifeFlight	Physician	Medical director
Canada	STARS	Physician	Chief medical officer
Canada	STARS	Nurse	Chief clinical officer
Finland	FinnHEMS	Physician	Clinician
New Zealand	Northern Rescue Helicopter	Physician	Clinician/Medical director
Norway	Norwegian Air Ambulance	Physician	Clinician/Director of research
Wales	EMRTS Cymru	Paramedic	Clinician
Wales	EMRTS Cymru	Physician	Clinical informatics and research manager

BCEHS: British Columbia Emergency Health Services; EMRTS: Emergency Medical Retrieval and Transfer Service; HEMS: Helicopter Emergency Medical Services; RFDS: Royal Flying Doctor Service; RSQ: Retrieval Services Queensland; STARS: Shock Trauma and Rescue Services

### Deployment

Several studies described significant operational and logistical challenges associated with providing equity of care across vast access- and weather- challenged geography [20–26]. The Australian and New Zealand College of Anaesthetists (ANZCA) guideline for the transport of critically ill patients and the Intensive Care Society (ICS) and Faculty of Intensive Care Medicine (FICM) guidance on the transfer of the critically ill adult [27, 28] both described how the mode of transport should balance the nature of the illness, the urgency of transfer, distance, availability of transport, mobilization times, geography, traffic, weather conditions and cost. Furthermore, the ICS, FICM and ANZCA [27, 28] all identified the importance of central coordination of missions and real-time clinician support. Most SMEs describe central coordination with clinician (physician or paramedic) support.

An analysis of 1124 adult critical care transfers in the South West Critical Care Network in England found that ad-hoc transfers did not meet the standard for training, clinical experience, or timeliness compared to transfers with dedicated transport teams [29]. Furthermore, a study in Sweden found that transferring patients between ICUs transfers during periods of demand–supply mismatch was associated with an increased mortality rate [30]. A recent study from Norway explored the concept of introducing fairness in air ambulance base location planning and the importance of looking for optimal solutions [31].

All SMEs interviewed described clear mission statements, mission profiles and deployment priorities including 24/7/365 coverage. All SMEs identified varying abilities to transport special populations (neonatal, pediatric, obstetrical, bariatric, psychiatric, and extracorporeal life support [ECLS]) with bariatric, psychiatric and ECLS patients being the most challenging. Most SMEs described specialized hospital-based transport teams for neonatal and pediatric transports. Ornge utilized dedicated ground critical care and specialized mental health teams. Royal Flying Doctor Service (RFDS) Queensland used a specialized ambulance for all bariatric and ECLS patients, and the Norwegian Air Ambulance used on-call HEMS physicians with specialty training for all ECLS transports. FinnHEMS had limited special population capability, with most of these types of patients transported by ground using ad-hoc teams.

*Common Theme(s): Clearly defined mission statements, mission profiles and deployment priorities with 24/7/365 response capacity. Central coordination with clinician support. Use of dedicated or specialized transport teams*.

### Crew composition

In 2011, Jashapara [32] proposed the optimal, most productive, and cost-effective solution to enhance prehospital critical care capacity in the United Kingdom was by using specialized (critical care) paramedics, with physicians providing medical support, clinical governance, and advice as part of a multi-professional team approach. This report has subsequently been criticized for drawing these conclusions from inappropriate or unsound methods and likely delayed the implementation of critical care paramedics in the United Kingdom by some years [33]. Several studies demonstrated that with extensive training, education, exposure to high-acuity patients and physician support, critical care or intensive care paramedics could successfully perform complex procedures in the prehospital and transport setting [21, 34, 35]. Similarly, Denton et al. [36] established that advanced critical care practitioners could provide an alternative care process for critically ill adults who require external transfer. Venter et al. [37] found that patient safety could be improved by using dedicated crew with additional training. Laverty et al. [38] found a trend toward decreased mortality in trauma patients treated by more experienced or advanced providers. Corfield et al. [39] concluded that the combined skills of a physician and paramedic retrieval team offer potentially life-saving benefits for patients with critical illness and injuries in rural and remote Scotland. Moors et al. [40] found that physician-based HEMS was associated with an additional 2.5 lives saved per 100 dispatches for severely injured pediatric patients. Smith et al. [41] demonstrate an additional 3.22 survivors per 100 severe trauma patients when treated by a physician and critical care paramedic team. Maddock et al. and Lyons et al. [42, 43] demonstrated that the physician and critical care practitioner model was associated with a reduction in 30-day mortality for patients with blunt traumatic injury compared with care provided by ground paramedics. Hesselfeldt et al. [44] observed a significant reduction in time to the trauma center for severely injured patients and reduced proportions of secondary transfers with physician-led HEMS. Conversely, Hepple et al. [45] found no significant evidence of survival benefit associated with physician-led HEMS and Masterson et al. [46] concluded that HEMS confers a patient benefit regardless of crew composition.

All SMEs described a more advanced scope of practice than ground paramedics with 24/7/365 online acute care physician support linked to standard operating procedures and clinical practice guidelines with regular and ad-hoc review points. Most SMEs reported considerable variation in crew composition as shown in Table 4.

**Table 4:** Crew composition

Location	Organization	Composition				
		Mission Specific	Physician	Paramedic^2^	Nurse	Respiratory Therapist
Australia	Air Ambulance Victoria	✓	✓	✓		
Australia	Greater Sydney Area HEMS		✓	✓		
Australia	Retrieval Services Queensland	✓	✓	✓	✓	
Australia	RFDS Queensland^1^	✓	✓		✓	
Canada	BCEHS			✓		
Canada	EHS LifeFlight	✓		✓	✓	✓
Canada	Ornge			✓		
Canada	STARS	✓	✓	✓	✓	
Finland	FinnHEMS	✓	✓	✓		
New Zealand	Northern Helicopter Rescue	✓	✓	✓		
Norway	Norwegian Air Ambulance		✓	✓		
Wales	EMRTS Cymru^3^	✓	✓	✓	✓	

BCEHS: British Columbia Emergency Health Services; EMRTS: Emergency Medical Retrieval and Transfer Service; HEMS: Helicopter Emergency Medical Services; RFDS: Royal Flying Doctor Service; RSQ: Retrieval Services Queensland; STARS: Shock Trauma and Rescue Services; 1. The majority were single nurse retrievals; 2. Includes primary, advanced, and critical care paramedics; 3. EMRTS Cymru utilizes a critical care practitioner with either a paramedic or nursing background

*Common Theme(s): Advanced scope of practice with 24/7/365 online acute care physician support linked to standard operating procedures and clinical practice guidelines*.

### Crew selection and education

Most prehospital or retrieval physicians are either senior registrars or consultants and typically come from anaesthesia, emergency medicine or intensive care backgrounds [42, 46] while paramedics typically undertake intensive multi-year process to become a critical or intensive care paramedic [21, 22, 47, 48]. Most SMEs outline a rigorous selection process including a minimum of five years’ experience at an advanced license level, knowledge-based and clinical performance assessments, and scenario-based interviews. Air Ambulance Victoria and Greater Sydney Area HEMS also described medical, physical, and psychological testing during their selection processes. While the Norwegian Air Ambulance mostly recruited paramedics from special operations forces.

*Common Theme(s): Rigorous selection process of candidates*.

A recent study identified many similarities among independently developed prehospital and retrieval medicine courses worldwide including the use of lectures, simulation and discussion groups but also noted some important differences including variations in content and delivery methods based on participant background and patient population [49]. Most SMEs described considerable variation in the length, structure, and delivery method of initial education, with a service-sponsored university-based graduate education model being the most common. Continuing medical education varied but was typically monthly, consisting of a combination of low- and high-fidelity simulations of clinical skills, case reviews, and quarterly education and training.

*Common Theme(s): Service-sponsored university-based education model*.

### Clinical governance

Several studies described models of clinical governance. These models often included monthly clinical governance days, thorough case reviews and comprehensive registry audits [47, 48, 50–52]. Most SMEs reported the use of daily simulations, review of all recent patient transports, monthly case reviews, morbidity, and mortality rounds, and quarterly or yearly specialized education and recertification training. However, all systems without an advanced clinical governance model consider their lack to be a significant weakness.

*Common Theme(s): Advanced clinical governance*.

### Quality assurance and quality improvement

There is paucity of literature describing quality assurance and quality improvement in CCT or retrieval medicine. In 2017, The EQUIPE-collaboration group reached consensus on 15 response specific and 11 system specific quality indicators for physician-staffed emergency medical services encompassing all six quality dimensions outlined by the Institutes of Medicine, although these still need to be prospectively tested for feasibility, validity and reliability in clinical datasets [53]. Similarly Ornge [51] outlined a framework of quality assurance and quality improvement initiatives for improving patient experience, clinical practice, and operational service delivery to meet the transport needs of residents in Ontario, Canada within a broader healthcare system. Another study established a benchmark for audit and quality improvement of advanced critical care practitioner-led interfacility transport [36]. Lastly, a recent scoping review by Edwards et al. [54] resulted in the development of a quality framework comprised of eight outcomes including: asset and team type, access to definitive interventions, prehospital factors, mortality, morbidity, the responsiveness of service, accessibility of service and patient disposition. If adopted, this framework could allow for better comparison between systems.

Researchers in Scandinavia have demonstrated the feasibility of gathering detailed and comprehensive data nation-wide on all HEMS missions and treated patients using centralized and bespoke databases and data entry templates [55–57]. They also indicated that a national database is an important data source for research and quality improvement and could provide valuable insights into where HEMS operations could be improved [55–57]. Similarly, Greater Sydney Area HEMS described the long-term effectiveness of their airway registry on the completeness of documentation of prehospital rapid sequence intubation [58]. They also highlighted the importance of collecting variables, as proposed in the literature, when establishing a registry. Furthermore, they emphasized that time should be invested in designing a data entry template to minimize free-text fields and unambiguous response options. Significant variables should be recorded through mandatory fields to ensure compliance [58].

*Common Theme(s): Use of a comprehensive database to guide quality assurance and quality improvement*.

Most SMEs describe advanced quality assurance and quality improvement programs, including equipment standardization, the use of checklists, regular case reviews and registry audits (Table 5). All systems without a robust quality assurance and quality improvement program viewed this lack as a significant weakness.

**Table 5:** Quality assurance and quality improvement

Location	Organization	Quality assurance/quality improvement
Australia	Air Ambulance Victoria	Safer Care Victoria, multiple state and national registries, equipment standardization, routine use of checklists, and monthly clinical governance days including case reviews and various audits
Australia	Greater Sydney Area HEMS	Anesthesia (rapid sequence induction [RSI]), transfusion and ultrasound audits, clinical excellence commission, equipment standardization, routine use of checklists, and physician follow-up for all transported patients
Australia	Retrieval Services Queensland	Quality management system, clinical incident management system, various monthly clinical audits, and equipment standardization
Australia	RFDS Queensland	Mandatory reporting and review of “high-interest events” withing 24 h, quality assurance committee reporting to Queensland Health, bimonthly case reviews, and state-wide aeromedical meeting
Canada	BCEHS	Airway registry
Canada	EHS LifeFlight	Airway registry and transfusion audits
Canada	Ornge	Anesthesia (RSI), CCT and transfusion audits, and equipment standardization
Canada	STARS	Review of all ePCRs by either the base physician or senior clinician; airway and transfusion audits, and equipment standardization
Finland	FinnHEMS	FinnHEMS database was established to record detailed information on all HEMS missions in the country
New Zealand	Northern Helicopter Rescue	Ground Air Medical qUality Transport (GAMUT) quality improvement collaborative, and anesthesia (RSI), transfusion, and ultrasound audits
Norway	Norwegian Air Ambulance	Anesthesia (RSI), CCT and transfusion audits
Wales	EMRTS Cymru	Anesthesia (RSI and procedural sedation), CCT and transfusion audits, equipment standardization, and routine use of checklists

BCEHS: British Columbia Emergency Health Services; EMRTS: Emergency Medical Retrieval and Transfer Service; HEMS: Helicopter Emergency Medical Services; RFDS: Royal Flying Doctor Service; RSQ: Retrieval Services Queensland; STARS: Shock Trauma and Rescue Services

*Common Theme(s): Equipment standardization including pre-packaged and sealed equipment, pre-drawn high-use medications, and the use of checklists*.

### Research

There are well documented challenges associated with conducting prehospital and transport medicine research, including the extensive clinical, ethical, and logistical factors limiting clinical trials and accurate data collection and the inherent heterogeneity of the patient populations and systems that allow for generalization of results. [57, 59].

All SMEs reported varying degrees of research capacity, with the Norwegian Air Ambulance Foundation leading the way in out-of-hospital research with 31 doctoral candidates, 19 other researchers and six active randomized control trials. Air Ambulance Victoria, and Emergency Medical Retrieval and Transfer Service (EMRTS) Cymru both described a variety of ongoing base-specific research trials.

*Common Theme(s): Organizational prioritization and support for research*.

## Discussion

A high-performance CCT or retrieval system is essential to support the equity of care and overcome the tyranny of distance for those living in rural, remote, and indigenous communities. Our mixed-methods study used novel methods to integrate quantitative data from a narrative review of the literature and qualitative data from semi-structured interviews to provide a better understanding of the current practices of government and charity funded CCT or retrieval systems.

By exploring the characteristics of similarly funded and administered systems we can potentially improve the effectiveness of current practices. This study revealed a paucity of literature describing the clinical, operational, and educational best practices of government or charity funded CCT and retrieval systems. Most of the published literature is retrospective, from Northern Europe and focuses on prehospital critical care rather than CCT or retrieval medicine, which limits the generalisability of their conclusions. However, when quantitative findings from the literature review were integrated with the qualitative insights from the semi-structured interviews with identified SMEs, several common themes emerged including clearly defined mission profiles, central coordination, rigorous selection processes, service-sponsored graduate education, use of dedicated or specialized teams, advanced clinical governance, equipment standardization, comprehensive data set and organization commitment to research (Table 6). These common themes could form the basis of best of practice.

**Table 6:** Common themes

1	Clearly defined mission statements, mission profiles and deployment priorities with 24/7/365 response capacity
2	Central coordination with clinician (physician or paramedic) support
3	Use of dedicated or specialized transport teams
4	Advanced scope of practice with 24/7/365 online acute care physician support linked to standard operating procedures and clinical practice guidelines
5	Rigorous selection process of candidates
6	Service-sponsored university-based education model
7	Advanced clinical governance
8	Use of a comprehensive database to guide quality assurance and quality improvement
9	Equipment standardization including pre-packaged and sealed equipment, pre-drawn high-use medications, and the use of checklists
10	Organizational prioritization and support for research

The literature described considerable variation and little consensus regarding crew composition worldwide and is largely influenced by practitioner availability, specific patient need, funding, historical practice, interpretation of the evidence base and the needs of the larger health care system. While most organizations in this study utilize multidisciplinary teams, the literature only identified the use of dedicated or specialized transport teams as best practice.

Clinical governance is a framework for accountability and decision-making that provides the foundation for quality improvement [60]. Some examples of clinical governance identified include daily simulations of high-acuity low-occurrence (HALO) skills, daily peer review of high-interest cases or events, daily review of all patient transports for clinical competency and documentation, monthly clinical governance days including case reviews and audits These initiatives demonstrate the importance of continuous quality improvement within CCT or retrieval systems.

The quality of data is a well-reported challenge of prehospital and transport research. Addressing these challenges can improve performance evaluation, quality assurance, and potential collaboration. High-quality datasets, such as those described by Danish, Finnish, and Greater Sydney Area HEMS, could enable direct service-level comparison and facilitate higher-quality research.

### Limitations

The rapid review of the literature was conducted by a single author (AG) and is therefore at risk of selection and data extraction bias. These risks were minimized with the assistance of a medical librarian and by utilizing clearly defined inclusion and exclusion criteria. The search strategy was limited by date, language, and available full text; therefore, relevant evidence could have been excluded. Furthermore, articles were excluded if they primarily described private, for-profit, or military programs. Most importantly, this review was limited by the volume and quality of the available evidence, which reduced the generalisability of their conclusions. The semi-structured interviews were limited by time, and the availability of identified SMEs, as some interview requests went unanswered.

## Conclusion

This mixed-methods study underscores the paucity of literature describing current clinical, operational, and educational practices of government or charity-funded CCT or retrieval programs operating across access- and weather- challenged geography. While many common themes were identified including clearly defined mission profiles, use of dedicated or specialized transport teams, central coordination, rigorous selection processes, service-sponsored graduate education, and strong clinical governance, there is little consensus and considerable variation in current practices. Further research is needed to define and harmonize best practices within the CCT and retrieval environments worldwide.

### Supplementary Information

**Additional file 1.** Appendix 1.

## Data Availability

Not applicable.

## Abbreviations

ANZCA: Australian and New Zealand College of anaesthetists
CAMTS: Commission for the accreditation of air medical transport systems
CCT: Critical care transport
ECLS: Extracorporeal life support
EMRTS: Emergency medical retrieval and transfer service
EURAMI: European aero-medical institute
FICM: Faculty of intensive care medicine
HALO: High-acuity low-occurrence
HEMS: Helicopter emergency medical system
ICS: Intensive care society
SME: Subject matter expert
