# Supplementary material for: Overcoming distance: an exploration of current practices of government and charity-funded critical care transport and retrieval organizations
Source: Scand J Trauma Resusc Emerg Med. 2023 Oct 3;31:52. doi: 10.1186/s13049-023-01125-6 (PMC10548638; doi:10.1186/s13049-023-01125-6)
Supplement: Supplementary file 1 — Additional file 1. Appendix 1. [file 13049_2023_1125_MOESM1_ESM.docx]

**Appendix 1.**

| CENTRAL | (critical care prehospital or trauma or intensive care or paramedic or flight paramedic or critical care paramedic or critical care practitioner or flight nurse or physician or doctor):ti,ab,kw AND (Transport or aircraft or airplane or fixed-wing or helicopter or helicopter emergency medical services or HEMS or rotary-wing or retrieval or ambulance or air ambulance or air-medical or air-medicine services or aeromedical):ti,ab,kw AND (Evaluation or service evaluation or service delivery or review or system review or report or experience or lesson or policy or quality or quality indicators or quality assurance or quality improvement or QA or QI or study or observational):ti,ab,kw |
| --- | --- |
| OVID EMBASE | (“critical Care” OR “prehospital” OR “trauma” OR “intensive care” OR “paramedic” OR “flight paramedic” OR “critical care paramedic” OR “critical care practitioner” OR “flight nurse” OR “physician” OR “doctor”) AND (“Transport” OR “aircraft” OR “airplane” OR “fixed-wing” OR “helicopter” OR “helicopter emergency medical services” OR “HEMS” OR “rotary-wing” OR “retrieval” OR “ambulance” OR “air ambulance” OR “air-medical” OR “air-medicine services” OR “aeromedical”) AND (“Evaluation” OR “service evaluation” OR “service delivery” OR “review” OR “system review” OR “report” OR “experience” OR “lesson” OR “policy” OR “quality” OR “quality indicators” OR “quality assurance” OR “quality improvement” OR “QA” OR “QI” OR “study” OR “observational”).mp. [mp=title, abstract, heading word, drug trade name, original title, device manufacturer, drug manufacturer, device trade name, keyword heading word, floating subheading word, candidate term word] |
| Ovid MEDLINE | (“critical Care” OR “prehospital” OR “trauma” OR “intensive care” OR “paramedic” OR “flight paramedic” OR “critical care paramedic” OR “critical care practitioner” OR “flight nurse” OR “physician” OR “doctor”) AND (“Transport” OR “aircraft” OR “airplane” OR “fixed-wing” OR “helicopter” OR “helicopter emergency medical services” OR “HEMS” OR “rotary-wing” OR “retrieval” OR “ambulance” OR “air ambulance” OR “air-medical” OR “air-medicine services” OR “aeromedical”) AND (“Evaluation” OR “service evaluation” OR “service delivery” OR “review” OR “system review” OR “report” OR “experience” OR “lesson” OR “policy” OR “quality” OR “quality indicators” OR “quality assurance” OR “quality improvement” OR “QA” OR “QI” OR “study” OR “observational”).mp. [mp=title, book title, abstract, original title, name of substance word, subject heading word, floating sub-heading word, keyword heading word, organism supplementary concept word, protocol supplementary concept word, rare disease supplementary concept word, unique identifier, synonyms, population supplementary concept word, anatomy supplementary concept word] |
| PubMed | (“critical Care"[Mesh] OR “prehospital”[Title/Abstract] OR “trauma”[Title/Abstract] OR “intensive care”[Title/Abstract] OR “paramedic”[Title/Abstract] OR “flight paramedic”[Title/Abstract] OR “critical care paramedic”[Title/Abstract] OR “critical care practitioner”[Title/Abstract] OR “flight nurse”[Title/Abstract] OR “physician”[Title/Abstract] OR “doctor”[Title/Abstract]) AND (“Transport "[Mesh] OR “aircraft”[Title/Abstract] OR “airplane”[Title/Abstract] OR “”[Title/Abstract] “fixed-wing”[Title/Abstract] OR “helicopter”[Title/Abstract] OR “helicopter emergency medical services”[Title/Abstract] OR “HEMS”[Title/Abstract] OR “rotary-wing”[Title/Abstract] OR “retrieval”[Mesh] OR “ambula*"[Title/Abstract] OR “air ambulance”[Title/Abstract] OR “air-medical”[Title/Abstract] OR “air-medicine services”[Title/Abstract] OR “aeromedical”[Title/Abstract]) AND (“Evaluation”[Mesh] OR “service evaluation”[Title/Abstract] OR “service delivery”[Title/Abstract] OR “review”[Title/Abstract] OR “system review”[Title/Abstract] OR “report”[Title/Abstract] OR “experience”[Title/Abstract] OR “lesson”[Title/Abstract] OR “policy”[Title/Abstract] OR “quality”[Title/Abstract] OR “quality indicators”[Title/Abstract] OR “quality assurance”[Title/Abstract] OR “quality improvement”[Title/Abstract] OR “QA”[Title/Abstract] OR “QI”[Title/Abstract] OR “study”[Title/Abstract] OR “observational”[Title/Abstract]) |
